# Incidence, mortality and disability-adjusted life years of acute myocardial infarction in Kazakhstan: data from unified national electronic healthcare system 2014–2019

**DOI:** 10.3389/fcvm.2023.1127320

**Published:** 2023-08-02

**Authors:** Gulnur Zhakhina, Abduzhappar Gaipov, Alessandro Salustri, Arnur Gusmanov, Yesbolat Sakko, Sauran Yerdessov, Makhabbat Bekbossynova, Anara Abbay, Antonio Sarria-Santamera, Oguz Akbilgic

**Affiliations:** ^1^ Department of Medicine, Nazarbayev University School of Medicine, Astana, Kazakhstan; ^2^ Clinical Academic Department of Internal Medicine, CF “University Medical Center”, Astana, Kazakhstan; ^3^ National Research Cardiac Surgery Center, Astana, Kazakhstan; ^4^ Cardiovascular Section, Department of Internal Medicine, School of Medicine, Wake Forest University, Winston-Salem, NC, United States

**Keywords:** cardiovascular disease, acute myocardial infarction, risk factor, survival analysis, disability-adjusted life-years

## Abstract

**Background:**

Cardiovascular diseases contribute to premature mortality globally, resulting in substantial social and economic burdens. The Global Burden of Disease (GBD) Study reported that in 2019 alone, heart attack and strokes accounted for the deaths of 18.6 million individuals. Ischemic heart diseases, including acute myocardial infarction (AMI), accounted for 182 million disability-adjusted life years (DALYs) and it is leading cause of death worldwide.

**Aim:**

The aim of this study is to present the burden of AMI in Kazakhstan and describe the outcome of hospitalized patients.

**Methods:**

The data of 79,172 people admitted to hospital with ICD-10 diagnosis I21 between 2014 and 2019 was derived from the Unified National Electronic Health System and retrospectively analyzed.

**Results:**

The majority of the cohort (53,285, 67%) were men, with an average age of 63 (±12) years, predominantly of Kazakh (38,057, 48%) and Russian (24,583, 31%) ethnicities. Hypertension was the most common comorbidity (61,972, 78%). In males, a sharp increase in incidence is present after 40 years, while for females, the morbidity increases gradually after 55. Throughout the observation period, all-cause mortality rose from 101 to 210 people per million population (PMP). In 2019, AMI account for 169,862 DALYs in Kazakhstan, with a significant proportion (79%) attributed to years of life lost due to premature death (YLDs). Approximately half of disease burden due to AMI (80,794 DALYs) was in age group 55–69 years. Although incidence is higher for men, they have better survival rates than women. In terms of revascularization procedures, coronary artery bypass grafting yielded higher survival rates compared to percutaneous coronary intervention (86.3% and 80.9% respectively) during the 5-year follow-up.

**Conclusion:**

This research evaluated the burden and disability-adjusted life years of AMI in Kazakhstan, the largest Central Asian country. The results show that more effective disease management systems and preventive measures at earlier ages are needed.

## Introduction

Cardiovascular diseases (CVDs) continue to be one of the leading causes of premature mortality worldwide. According to the Global Burden of Disease (GBD) Study, in 2019, there were approximately 523 million prevalent cases of CVDs, constituting approximately 7% of the world population (1). Furthermore, statistics from the World Health Organization (WHO) indicate that more than three-quarters of cardiovascular disease-related deaths occur in low- and middle-income countries (2). While high-income countries have witnessed a decline in the incidence of acute myocardial infarction (AMI) and case-fatality rates associated to AMI (3), the increasing life expectancy, population growth, and aging population contribute to a heavier disease burden.

The quantifiable measure of fatal and non-fatal health consequences of diseases and injuries is a disability-adjusted life year (DALY). According to the data of the GBD Study, ischemic heart diseases accounted for 714,104 DALYs in Kazakhstan between 1990 and 2017 (4). Given these considerations, we aimed to assess the epidemiology of AMI in Kazakhstan, the largest Central Asian country.

## Materials and methods

### Study design and population

The data for this retrospective study obtained from the Unified National Electronic Health System (UNEHS) for the period of 2014–2019. Detailed information on the UNEHS and its databases can be found elsewhere (5). Patients included in the study were identified based on the International Classification of Diseases (ICD) codes, specifically those with the ICD-10 code of I21 (acute myocardial infarction) as the primary diagnosis. Additional information regarding the diagnostic criteria and protocols for patient management can be found in the Supplementary Table S1 (6).

**Figure 1:**
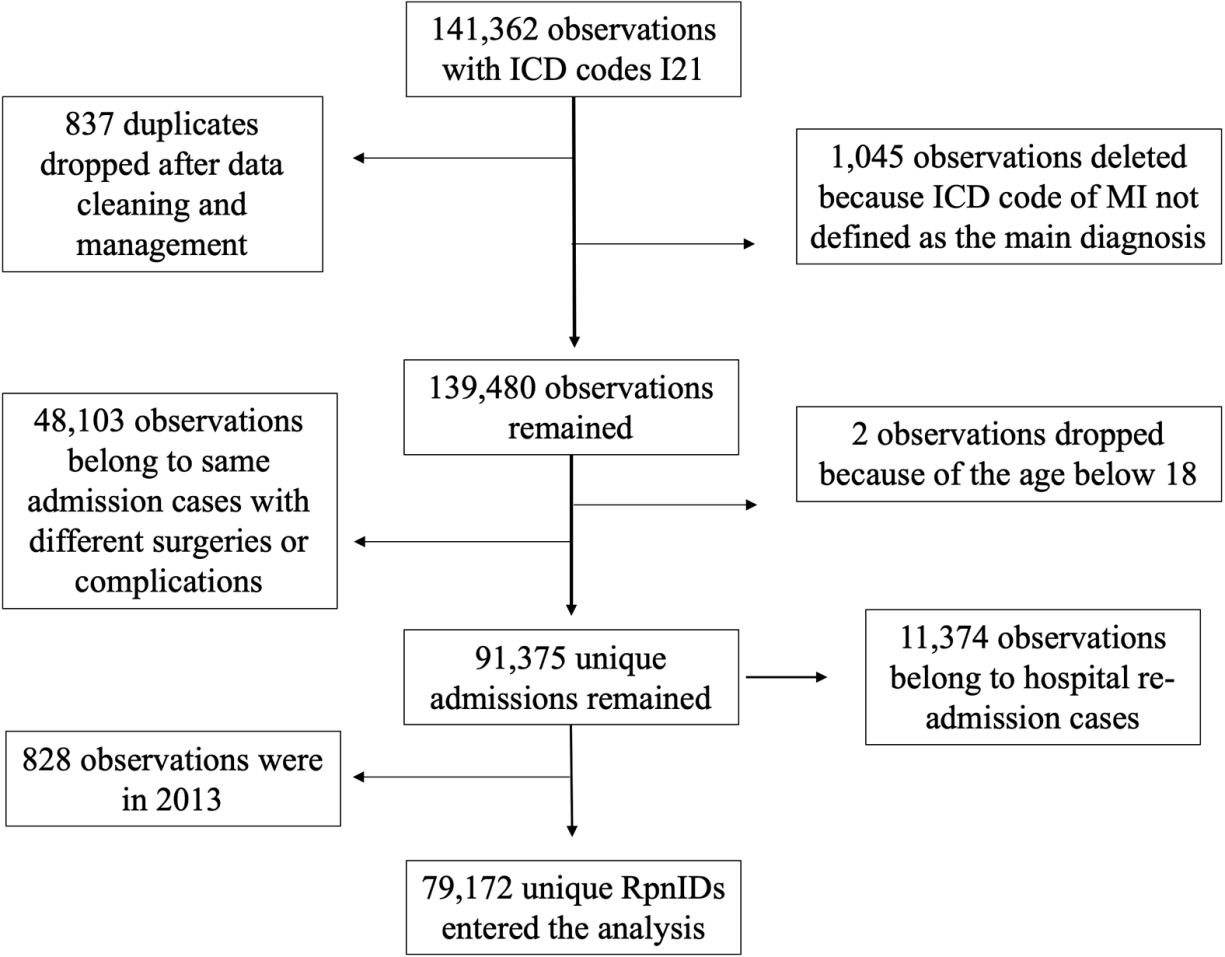
Flow chart diagram of cohort set-up.

From the initial pool of 162,099 records related to AMI, after thorough data cleaning and management, a cohort of 79,172 people with unique RPN IDs remained for the analysis. Only information pertaining to the first-ever occurrence of AMI during the observation period was considered for analysis. Detailed information on cohort setup is presented in Figure 1.

Population numbers were obtained from the Statistics Committee of the Ministry of National Economy of the Republic of Kazakhstan. In 2021, the population of Kazakhstan was over 18 million people. Considering the gender composition, the percentage of males in the population (48.5%) was slightly lower than that of females. The detailed information on population size for each gender and age group is given in Supplementary Table S2.

### Exposures and covariates

The analysis included information on the date of birth, gender, ethnicity, living area, social status, date of hospital admission, date of hospital discharge, and date of death, if applicable. The birth date and death date were obtained from the Population Registry. Age was categorized as following: (1) below 34 years old, (2) 35–50 years old, (3) 51–70 years old, and (4) above 70 years old. In Kazakhstan, there are over 120 nationalities, with a significant prevalence of Kazakh followed by Russians and various other minority groups. Therefore, ethnicity was classified into Kazakhs (*n* = 38,397), Russians (*n* = 24,897), and others (*n* = 16,707).

### Medical characteristics

Two primary revascularization procedures were examined in this study: coronary artery bypass graft surgery (CABG) (ICD-9 codes 36.10, 36.11, 36.12, 36.13, 36.14, 36.15, 36.16) and percutaneous coronary intervention (PCI) (ICD-9 codes 00.66, 36.01, 36.02, 36.06 and 36.07). The information on comorbidities such as cerebrovascular accident (CVA) (7), diabetes mellitus (DM) (8), hypertension (9), and chronic kidney disease (CKD) was collected by merging the databases using unique RPN IDs. All comorbid conditions were defined based on their respective ICD-10 codes.

### Outcome assessment

The study assessed the incidence, all-cause mortality, 30-day mortality, and in-hospital mortality in the cohort based on the hospital admission and discharge status of patients. The incidence rate (IR) represents the rate of first-ever events. Death occurring at any time during the observation period was accounted as all-cause death. The rates were calculated by dividing the absolute numbers by the total general population size of the general population at the end of each year. For the survival analysis, the start date was defined as the first day of the initial admission, and the follow-up period extended until December 31 of 2019, or until the day of death, if it occurred.

### Disability-adjusted life years calculation

DALY is a measure that combines the years of life lost due to premature death (YLLs) and years lived with disability (YLDs). The calculation follows the WHO methods for the global burden of disease estimates (10). The formula uses a simplified version of DALY, without age-weighting and time discounting.

The overall YLL was determined by summing the years of life lost for each age category group, ranging from 20 to 24 years old to 85+ years old. The number of deaths in each group was multiplied by the life expectancy at the age of death. According to the GBD 2010 reference life table, the life expectancy at birth for both males and females was 86.0 years (10).

The calculation of YLD follows a prevalence-based approach, as used by WHO and GBD 2010. The formula for YLD involves multiplying the prevalence of a condition by its corresponding disability weight (DW). In the case of AMI, the DW was found to be 0.432 during the first two days after the infarction episode and 0.074 from days 3 to 28 (10). The prevalence of AMI cases from 2014 to 2019 was multiplied by each DW, and the results were summed to obtain overall years lived with disability.

### Statistical analysis

All characteristics in the study are presented as categorical variables. Incidence and all-cause mortality rates, based on hospital admission and discharge status, were evaluated as both absolute numbers and rates per 1,000,000 population for each year of the observation period. Crude survival was demonstrated using Kaplan-Meier estimates, and the significance of difference was assessed using Log-rank tests.

Cox regression analyses were conducted after verifying the corresponding assumptions. Crude and adjusted hazard ratios were reported. Model 1 adjusted HR for socio-demographic factors such as age category, gender, and ethnicity. Model 2 included socio-demographic variables and comorbidities such as CVA, essential hypertension, DM, and CKD. Model 3 accounted for all the abovementioned factors, as well as revascularization strategies (CABG and PCI). The significance level was set at 0.05. All statistical analyses were performed using STATA 16.1.

The study utilized secondary data derived from the UNEHS. Since patients were not directly involved in the study, the requirement for informed consent from study participants was waived by the Nazarbayev University Institutional Review Ethics Committee (NU-IREC 490/18112021).

## Results

### Socio-demographic characteristics

The baseline characteristics of the cohort are presented in Table 1. During 2014–2019, a total of 79,172 people were admitted to the hospital due to acute myocardial infarction, with 33% being women and 67% men. Among the cohort, 68,628 (86%) were older than 50, and 42,052 (53%) were retired. Concurrent CVA, DM, hypertension, and CKD were present in 5%, 17%, 78%, and 12% of the cohort, respectively. The mortality rate per 100,000 patient-years in urban areas was calculated to be 20.4 [95% CI: 20.1–20.8], while in rural areas the corresponding rate was 17.4 [95% CI: 17.0–17.9], *p* < 0.001 (Table 1).

**Table 1:** Socio-demographic and medical characteristics of patients, who had MI between 2014 and 2019.

	Total (*n* = 79,172)	Alive (*n* = 61,155, 77%)	Dead (*n* = 18,017, 23%)	*p*-value	Mortality rate per 100,000 patient-years [95% CI]
Socio-demographics					
Gender, *n* (%)				<0.001	
Female	25,887 (33)	18,100 (30)	7,787 (43)		27.6 [27.0; 28.2]
Male	53,285 (67)	43,055 (70)	10,230 (57)		15.8 [15.5; 16.2]
Age category, *n* (%)				<0.001	
<34 years old	546 (0.7)	512 (0.8)	34 (0.2)		4.4 [3.2; 6.2]
35–50 years old	9,998 (13)	9,147 (15)	851 (4.8)		6.2 [5.8; 6.7]
51–70 years old	46,447 (58)	38,435 (63)	8,012 (44)		14 [13.7; 14.4]
>70 years old	22,181 (28.3)	13,061 (21.2)	9,120 (51)		42.8 [41.9; 43.7]
Ethnicity, *n* (%)				<0.001	
Kazakh	38,057 (48)	30,818 (50)	7,239 (40)		15.9 [15.6; 16.3]
Russian	24,583 (31)	17,612 (29)	6,971 (39)		25.1 [24.5; 25.7]
Other	16,532 (21)	12,725 (21)	3,807 (21)		19.5 [18.9; 20.1]
Living area, *n* (%)				<0.001	
Urban	53,350 (67)	40,722 (67)	12,628 (70)		20.4 [20.1; 20.8]
Rural	25,822 (33)	20,433 (33)	5,389 (30)		17.4 [17.0; 17.9]
Social status, *n* (%)				<0.001	
Employed	20,860 (26)	18,785 (31)	2,075 (12)		7.4 [7.1; 7.8]
Unemployed	11,216 (14)	9,651 (16)	1,565 (9)		10.9 [10.4; 11.5]
Retiree	42,052 (53)	28,665 (47)	13,387 (73)		29.8 [29.3; 30.3]
Disabled	2,014 (3)	1,523 (2)	491 (3)		26.1 [23.9; 28.5]
Other	3,030 (4)	2,531 (4)	499 (3)		13.4 [12.2; 14.6]
Comorbidities					
CVA, *n* (%)				<0.001	
Yes	4,329 (5)	2,472 (4)	1,857 (10)		40.3 [38.5; 42.2]
No	74,843 (95)	58,683 (96)	16,160 (90)		18.3 [18.1; 18.6]
Essential hypertension, *n* (%)				<0.001	
Yes	61,972 (78)	48,966 (80)	13,006 (72)		17.6 [17.3; 18.0]
No	17,200 (22)	12,189 (20)	5,011 (28)		26.3 [25.6; 27.0]
DM, *n* (%)				<0.001	
Yes	13,569 (17)	9,888 (16)	3,681 (20)		23.8 [23.0; 24.6]
No	65,603 (83)	51,267 (84)	14,336 (80)		18.6 [18.3; 18.9]
CKD, *n* (%)				<0.001	
Yes	9,539 (12)	6,724 (11)	2,815 (16)		26.5 [25.6; 27.5]
No	69,633 (88)	54,431 (89)	15,202 (84)		18.5 [18.2; 18.8]
Revascularization option					
CABG, *n* (%)				<0.001	
Yes	4,869 (6)	4,310 (7)	559 (3)		9.5 [8.8; 10.3]
No	74,303 (94)	56,845 (93)	17,458 (97)		20.1 [19.8; 20.4]
PCI, *n* (%)				<0.001	
Yes	42,256 (53)	35,381 (58)	6,875 (38)		13.5 [13.2; 13.8]
No	36,916 (47)	25,774 (42)	11,142 (62)		26.6 [26.1; 27.1]

CVA, cerebrovascular accident; DM, diabetes mellitus; CKD, chronic kidney disease; CABG, coronary artery bypass graft surgery; PCI, percutaneous coronary intervention.

**Figure 2:**
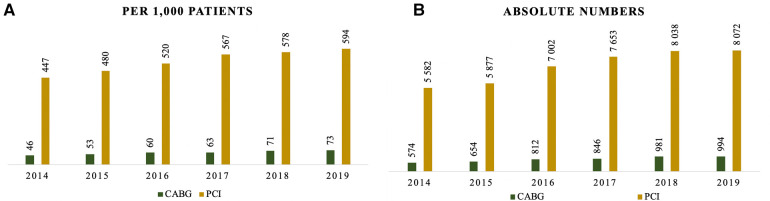
Frequency of cardiovascular surgeries by years: (**A**) per 1,000 patients; (**B**) absolute numbers.

During the hospital stay, 4,869 (6%) patients underwent CABG, while 42,256 (53%) had PCI (Table 1). The frequency of CABG per 1,000 patients was significantly lower compared to PCI during the observation period (Figure 2). Both interventions showed a gradual increase in rates from 2014 to 2019: CABG increased from 46 to 73 per 1,000 patients, while the rate for PCI escalated from 447 to 594 per 1,000 patients. Throughout the observation period, there were 5,956 (7.5%) in-hospital deaths and 7,121 (9%) deaths within 30 days.

### Incidence and mortality

**Figure 3:**
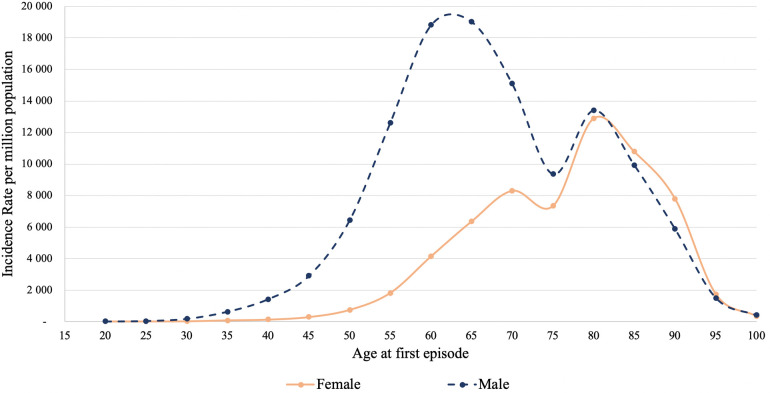
Age and sex-specific incidence rate of MI per 1,000,000 population for the years 2014–2019.

**Figure 4:**
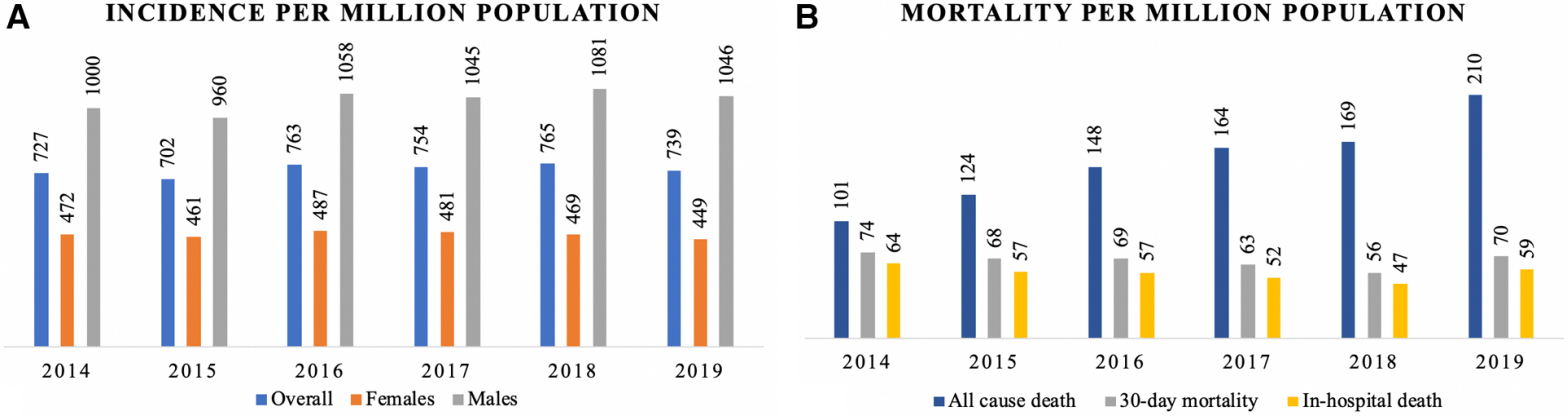
Burden of MI in Kazakhstan by years based on admission and discharge status: (**A**) incidence (overall, females, males) per 1,000,000 population; (**B**) mortality (all-cause, 30-day and in-hospital) per 1,000,000 population.

The age and sex-specific incidence rate (IR) among MI patients over the observation period is presented in Figure 3. According to the diagram, the IR among males rises dramatically till 60–65 years old; while for females, the growth is more gradual, peaking at 75–80 years of age. The IR based on hospital admission and discharge status did not change significantly over the observation period: 727 people per million population (PMP) in 2014 and 739 PMP in 2019 (Figure 4A). However, the all-cause mortality rate notably increased from 101 PMP in 2014 to 210 PMP in 2019 (Figure 4B).

### DALY

The life expectancy, years of life lost due to premature death (YLL), years lived with disability (YLD), and disability-adjusted life years (DALY) by age groups are presented in Table 2. In 2019, AMI accounted for 169,862.4 DALYs in Kazakhstan. The weight of premature death is significantly higher than the time lost due to ill health: 134,260.4 years of life lost (YLLs) vs. 35,602 YLDs, respectively. The highest burden of MI was observed in the 60–69-year-old age group, with an overall burden of 56,216.3 DALYs. There is a higher proportional contribution of YLLs to DALYs in all age groups, with the overall contribution being 79%. The highest burden of premature death and disability-adjusted life years is observed in the age group of 55–69 years, with 80,793.8 YLLs and 17,878.5 YLDs, respectively.

**Table 2:** Life expectancy, years of life lost due to premature death (YLLs) and years lived with disability (YLDs), and disability-adjusted life years (DALYs) by age groups in Kazakhstan over 2014–2019.

Age group	Life expectancy	YLL	YLD	DALY
18–24	68.85	63.9	17.2	81.1
25–29	58.94	353.6	62.7	416.3
30–34	54	540	221.6	761.6
35–39	49.09	1,620	562.7	2,182.7
40–44	44.23	3,626.9	1,361.1	4,988
45–49	39.43	7,097.4	2,726.3	9,823.7
50–54	34.72	11,770.1	4,603.1	16,373.2
55–59	30.1	18,120.2	6,437.3	24,557.5
60–64	25.55	21,564.2	6,223.8	27,788
65–69	21.12	23,210.9	5,217.4	28,428.3
70–74	16.78	15,706.1	3,080.5	18,786.6
75–79	12.85	18,131.4	3,062.3	21,193.7
80–84	9.34	9,031.8	1,499.3	10,531.1
85+	5.05	3,423.9	526.7	3,950.6
**Total**		**1,34,260**.**4**	**35,602**	**1,69,862**.**4**

### Survival analysis

**Figure 5:**
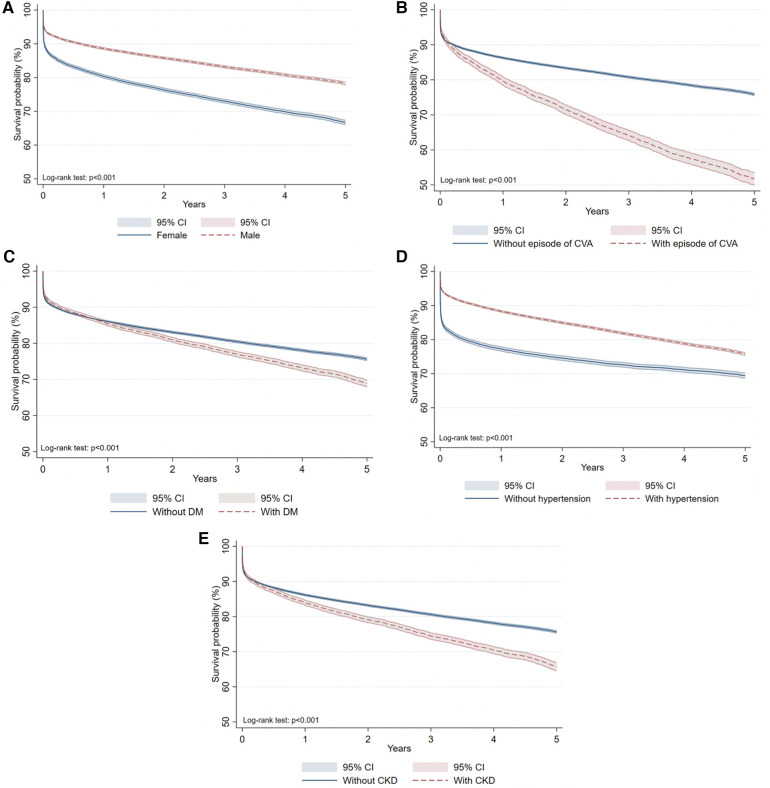
Kaplan-Meier survival curves due to all-cause mortality based on discharge status: (**A**) by gender; (**B**) by the history of CVA; (**C**) by DM comorbidity; (**D**) by hypertension comorbidity; (**E**) by CKD.

According to the survival analysis of AMI patients, males have better survival compared to females: 78.3% vs. 66.7% with a log-rank test *p*-value of <0.001 (Figure 5A). The crude 5-year survival of patients with a history of stroke is remarkably lower compared to patients without a history of stroke (51.8% and 75.9% respectively, *p* < 0.001) (Figure 5B). People without diabetes have higher survival rates compared to those with diabetes (75.6% and 68.8% respectively, *p* < 0.001) (Figure 5C). CKD is associated with decreased survival rate, showing a crude probability of 65.8% (Figure 5E). Opposite tendency is related to hypertension (Figure 5D).

### Hazard ratio by predictors

According to the Cox regression analysis in Table 3, males have a substantially lower risk of death [HR = 0.59, 95% CI: 0.57–0.61]; however, after adjustment for age and ethnicity, the difference becomes negligible [HR = 0.92, 95% CI: 0.89–0.94]. After adjustment for all sociodemographic factors, comorbidities, and revascularization procedures, Russians had a 37% higher risk of death compared to Kazakhs [HR = 1.37, 95% CI: 1.33–1.42]. The adjusted model showed that a history of stroke was associated with 84% [HR = 1.84, 95% CI: 1.75–1.93], diabetes with 29% [HR = 1.29, 95% CI: 1.24–1.34], and CKD with 45% [HR = 1.45, 95% CI: 1.39–1.51] higher risk of death in AMI patients. On the other hand, results show that hypertension was associated with 43% [HR = 0.57, 95% CI: 0.55–0.58] lower risk of death in the cohort. In terms of revascularization procedures, patients who underwent CABG had a lower risk of death by 57% [HR = 0.43, 95% CI: 0.39–0.47], and patients who underwent PCI had a lower risk of death by 44% [HR = 0.56, 95% CI: 0.54–0.57].

**Table 3:** Association between socio-demographic and medical parameters and all-cause mortality rates from MI for the years 2014–2019.

Variable	Unadjusted HR (95% CI)	*p*-value	Model 1 HR (95% CI)	*p*-value	Model 2 HR (95% CI)	*p*-value	Model 3 HR (95% CI)	*p*-value
Demographics								
Age category [<34 years old (ref)]								
35–50 years old	1.38 (0.99; 1.94)	0.060	1.39 (0.99; 1.95)	0.057	1.59 (1.13; 2.23)	0.007	1.77 (1.26; 2.48)	0.001
51–70 years old	3.01 (2.16; 4.19)	<0.001	2.91 (1.09; 4.05)	<0.001	3.46 (2.49; 4.84)	<0.001	3.77 (2.70; 5.25)	<0.001
>70 years old	8.42 (6.04: 11.7)	<0.001	7.74 (5.55; 10.8)	<0.001	9.16 (6.57; 12.8)	<0.001	9.18 (6.58; 12.8)	<0.001
Gender [Male vs. Female (ref)]	0.59 (0.57; 0.61)	<0.001	0.91 (0.89; 0.94)	<0.001	0.91 (0.88; 0.94)	<0.001	0.96 (0.93; 0.99)	0.005
Ethnicity [Kazakh (ref)]								
Russian	1.55 (1.51; 1.61)	<0.001	1.30 (1.26; 1.35)	<0.001	1.35 (1.29; 1.39)	<0.001	1.37 (1.33; 1.42)	<0.001
Other	1.22 (1.18; 1.27)	<0.001	1.05 (1.01; 1.09)	0.007	1.06 (1.01; 1.09)	0.005	1.06 (1.02; 1.11)	0.002
Comorbidities								
CVA	2.09 (1.99; 2.19)	<0.001			1.93 (1.84; 2.03)	<0.001	1.84 (1.75; 1.93)	<0.001
Essential hypertension	0.68 (0.65; 0.70)	<0.001			0.52 (0.51; 0.54)	<0.001	0.56 (0.55; 0.58)	<0.001
DM	1.26 (1.21; 1.30)	<0.001			1.27 (1.22; 1.31)	<0.001	1.29 (1.24; 1.34)	<0.001
CKD	1.39 (1.34; 1.45)	<0.001			1.43 (1.37; 1.49)	<0.001	1.45 (1.39; 1.51)	<0.001
Revascularization strategy [Only optimal medical treatment (ref)]								
CABG	0.33 (0.29; 0.35)	<0.001					0.43 (0.39; 0.47)	<0.001
PCI	0.46 (0.45; 0.48)	<0.001					0.56 (0.54; 0.57)	<0.001

CVA, cerebrovascular accident; DM, diabetes mellitus; CKD, chronic kidney disease; CABG, coronary artery bypass graft surgery; PCI, percutaneous coronary intervention.

Model 1: adjusted for demographics (age category, gender, ethnicity); Model 2: Model 1 + comorbidities (CVA, essential hypertension, DM, chronic kidney disease); Model 3: Model 2 + revascularization strategy (coronary artery bypass graft surgery, percutaneous coronary intervention).

## Discussion

This study evaluates the burden of AMI based on hospitalization records between 2014 and 2019 in Kazakhstan. In the cohort, the female-to-male ratio was 1:3, with the majority being Kazakhs, older than 50, and living in an urban area. A considerable proportion of the patients had hypertension, whereas stroke, diabetes, and CKD were not commonly observed. The incidence rate, 30-day death rate, and in-hospital death rate per million population did not change remarkably over the observation period, while all-cause mortality doubled. However, crude mortality among AMI patients decreased significantly during the follow-up time.

### Incidence of AMI based on hospitalization records

In Kazakhstan, between 2014 and 2019, the incidence rate of hospital admissions for AMI did not change significantly. In the Norwegian study based on national data from 2001 to 2014, researchers found that AMI rates declined by 2.7% per year over the observation period (11). It is important to note that the data used in this study only includes hospital admission and discharge reports, and therefore, it may not reflect the true incidence rate in the general population of Kazakhstan. It is worth considering that between 2011 and 2019, Kazakhstan implemented two State Programs for the Development of Healthcare, which placed significant emphasis on health education and promotion among the population (12, 13). The campaigns could have influenced the level of awareness about AMI in the community and led to improvements in the disease management system.

### Association of age and sex with AMI

According to hospital admission records, the incidence of AMI is significantly higher for men. This observation is consistent with findings from a Chinese prospective, nationwide, multicenter observational study conducted on patients diagnosed with AMI (14). Women, on the other hand, tend to be older at the time of the first AMI admission, and the incidence gradually increases after the age of 50, while for men, it rises after the age of 35. Similar patterns of age and sex-specific AMI occurrence rates have been observed in epidemiologic studies.

The lower risk of AMI in young women can be attributed to their pre- and peri-menopausal state and higher estrogen levels (15). Known risk factors for cardiovascular disease include family history of coronary diseases, smoking, diabetes, and obesity. The difference in age at which the first AMI occurs between males and females can be explained by variations in the occurrence of these factors over time between the two groups or the presence of healthier lifestyle patterns in women (16). Moreover, during the fertile period, high estrogen levels in females significantly reduce the risk of cardiovascular diseases compared to males of the same age (17).

This study identified two peaks of incidence for men. The first peak occurring at a younger age is consistent with findings from other research (18). However, the presence of the second peak for males at an older age is not observed in the existing literature. Further investigation and specific studies are required to address this issue and explore possible explanations. Nonetheless, these findings have implications for improving the recognition and timely management of infarction both in men and women. Implementing this knowledge can inform health policy decisions in this regard.

### Stroke and AMI

Age alone does not appear to be the primary reason for the high morbidity of AMI. As individuals age, they develop various comorbidities. This study specifically examined the impact of CVA, hypertension, DM, and CKD on the outcome of AMI patients. Stroke and AMI share common risk factors and are closely related in terms of their pathophysiology (19). The prevalence of CVA in this study cohort was found to be low. This finding is consistent with other studies that indicate a low likelihood of myocardial infarction occurring after a stroke (19). The lower occurrence of AMI after stroke can be attributed to preventive drug therapy administered following the initial cardiovascular event (20). However, individuals who experience CVD following a stroke have a lower survival probability (21), as observed in this study.

### Hypertension and AMI

Literature data regarding the association between essential hypertension and AMI are consistent. Elevated systolic and diastolic blood pressure contributes to the damage of blood vessels, increasing the risk of cardiovascular diseases. While essential hypertension is an independent risk factor for AMI, its combination with older age further elevates the morbidity of the illness (22). It is important to note that factors not considered in this research may have confounding effects and could potentially explain the finding of lower mortality rates among individuals with essential hypertension. Additionally, the inclusion of information on CVDs and CKDs, which are closely linked to hypertension, may influence the true effect of hypertension on AMI patients.

A cross-sectional study conducted among the Kazakhstani population revealed that a majority of hypertensive individuals were taking antihypertensive medications (23), indicating a potentially high rate of blood pressure control. The existing literature consistently highlights the strong association between hypertension and adverse outcomes (22). Further research should focus on investigating the impact of medication, lifestyle changes, and the reduction of modifiable risk factors reduction in relation to hypertension and its outcomes.

### Diabetes mellitus and AMI

Diabetes mellitus is recognized as another risk factor for AMI. The results of this study are in line with previous research, indicating higher mortality rates among diabetic patients (24). Interestingly, after adjusting for all socio-demographic factors, the mortality rate for diabetic patients was found to be less than 30%, which contrasts with earlier research conducted before 2010 that reported a probability of adverse outcomes exceeding 70% (25). The advancements in diagnostic tools, medical treatments, and healthcare management systems may have played a role in improving the life of individuals with diabetes.

### CKD and AMI

One additional comorbidity examined in this research is CKD at any stage. The prevalence of renal dysfunction in the cohort was found to be 12%, which aligns with findings from other studies that have utilized large health administrative datasets (26). Patients with CKD are known to have an increased risk of atherosclerosis and higher mortality rates following acute myocardial infarction. Due to the kidney dysfunction and the heightened likelihood of infections or organ failure, AMI patients with CKD may not receive invasive treatments such as CABG or PCI (27). Consequently, the long-term survival rates of AMI patients with CKD tend to be low. It is important to conduct separate studies to investigate the efficacy of invasive therapies specifically for this high-risk group, in order to enhance the healthcare management system.

### Mortality among the AMI population

The results of this study indicate a significantly higher mortality rate among Russians compared to Kazakhs. However, it is challenging to attribute this difference in survival solely to race. Other social determinants, including education level, employment status, socioeconomic situation, and cultural disparities in lifestyle and dietary habits, should be taken into account. In the population-based study conducted by Sharygin and Gulliot, it was shown that Russians have higher adult mortality rates than Central Asians, even after adjusting for socioeconomic status (28). The mortality gap in that study was attributed to an increased risk of death associated with alcohol consumption.

The development of cardiovascular services in Kazakhstan was relatively delayed, with significant advancements occurring in the 2010s (12, 13). Throughout the observation period, there was a threefold increase in human resources and technical equipment, which positively impacted the survival of AMI patients. In terms of mortality in Kazakhstan, it is noteworthy that the country has implemented a national guideline for the diagnosis and treatment of acute myocardial infarction (AMI) (6). This guideline aligns with the diagnostic algorithms endorsed by the international cardiology communities (29, 30). Early diagnosis and intervention of AMI cases are crucial for improving outcomes, and the implementation of early screening programs holds great significance. These initiatives aim to enhance the timely identification and management of AMI, ultimately leading to improved patient outcomes and reduced mortality rates. Despite improvements in the disease management system over the observation period, it is important to consider that higher life expectancy and an aging population could contribute to increased mortality rates from AMI in the later years.

### Disability-adjusted life years

Myocardial infarction is an acute disease that carries an elevated risk of cardiac dysfunction for approximately a month (31). The cardiovascular system is primarily damaged in the first two days after initial symptoms, resulting in disability, and the rest of the month is needed to restore normal cardiac functioning (32). Therefore, the contribution of years lived with a disability to the total DALY is low. It is not appropriate to compare the burden of AMI in terms of disability-adjusted life years between Kazakhstan and other countries without proper country-level adjustments on population size, cohort set-up, and observation period. In this study, DALYs for 50–69 years old people were twice as high as those individuals older than 70. In comparison, a Portuguese study reported a higher DALY burden for the latter group. This finding is significant considering that the proportion of elderly individuals and the median age are higher in Portugal (33, 34).

### Revascularization strategies for AMI

The results of this study revealed a significant decrease in the crude mortality rate among AMI patients over the observation period. This finding is consistent with the impact of Kazakhstani healthcare programs (12, 13) that have focused on improving the management of cardiovascular diseases. These programs have prioritized the development of heart and stroke centers, reducing CV mortality, and enhancing cardiovascular services (35). This research primarily focused on two revascularization interventions, namely PCI and CABG, which aim to restore adequate blood supply through the cardiovascular system. Given that the development of cardiovascular services in Kazakhstan is a relatively recent phenomenon, there have been variations in the accessibility of these procedures and the overall management of AMI patients. Consequently, the frequency of PCI was notably higher compared to CABG. Although the unadjusted model did not demonstrate a significant difference between the two, after adjusting for sociodemographic factors and comorbidities, CABG proved to be more effective in reducing mortality.

Previous studies have elucidated this finding by highlighting that myocardial infarction is often associated with non-flow-limiting stenosis, which is effectively addressed by CABG (36), whereas PCI is more suitable for treating flow-vessel occlusions (37). Furthermore, a recent systematic review evaluated randomized clinical trials comparing these interventions and concluded that CABG provides benefits in terms of protection against new myocardial infarctions (38). However, considering the difference in medical facility levels depending on hospital location, patient characteristics, and access to revascularization, intervention strategies should be examined separately to accurately assess their impact on reducing mortality rates.

### Strengths and limitations

The evaluation of epidemiology and the burden of disease in this study has several advantages. Firstly, it provides a comprehensive overview of acute myocardial infarction in Kazakhstan. Large cohort studies assessing the incidence, mortality, and disability-adjusted life years of AMI using nationwide databases in Central Asian countries are scarce, making these findings particularly valuable. They can contribute to the development of improved protocols and strategies for managing the disease in healthcare settings, taking into account socio-demographic factors and cultural differences. In addition, it may increase community awareness campaigns and raise concerns about a healthy lifestyle to avoid early AMI morbidity. Finally, the results of this study may stimulate further research on the cost-effectiveness of surgical interventions and the management of AMI management to evaluate the economic burden of disease.

Despite the aforementioned advantages, there are several limitations. First of all, the use of hospitalization records only captures a subset of AMI cases, and it does not account for pre-hospital morbidity and mortality. This means that the true incidence and mortality rates of AMI may be underestimated since cases occurring outside of the hospital setting are not included. This is particularly relevant for the older age groups, where a significant proportion of AMI cases may not result in hospitalization. Additionally, the database used in this study does not provide information on important factors such as tobacco use, blood cholesterol or triglyceride levels, congestive heart failure, arrhythmias, obesity, family history of heart attack, stress levels, illicit drug use, and autoimmune conditions, which are known to be comorbidities that can impact survival in AMI patients. The absence of these data limits the ability to fully understand the factors influencing outcomes in this population. Furthermore, errors during disease coding are possible, which could introduce inaccuracies in the analysis. The lack of cause of death information is also a limitation, as it restricts the ability to calculate cause-specific mortality rates and understand the specific factors contributing to mortality in AMI cases. These limitations should be taken into account when interpreting the findings of the study and further research should aim to address these gaps to provide a more comprehensive understanding of AMI in the population.

## Conclusion

This is the first study to evaluate the incidence, mortality, and disability-adjusted life years of AMI in Kazakhstan. The current study retrospectively analyzed nationwide admission and discharge data of myocardial infarction cases between 2014 and 2019. The incidence and mortality rates per million population did not change significantly over the observation period. However, there was a noticeable decrease in the crude mortality rate among AMI patients during the follow-up period, indicating effective management of hospitalized patients. Further educational campaigns targeting disease prevention and reduction of risk factors are warranted.

## Data Availability

The data analyzed in this study is subject to the following licenses/restrictions: The data that support the findings of this study are available from the Republican Center for Electronic Health of the Ministry of Health of the Republic of Kazakhstan but restrictions apply to the availability of these data, which were used under license for the current study, and so are not publicly available. Data are however available from the corresponding author, Gaipov A., upon reasonable request and with permission of the Ministry of Health of the Republic of Kazakhstan. Requests to access these datasets should be directed to Abduzhappar Gaipov, abduzhappar.gaipov@nu.edu.kz.
