# Supplementary material for: Incidence, mortality and disability-adjusted life years of acute myocardial infarction in Kazakhstan: data from unified national electronic healthcare system 2014–2019
Source: Front Cardiovasc Med. 2023 Aug 2;10:1127320. doi: 10.3389/fcvm.2023.1127320 (PMC10433224; doi:10.3389/fcvm.2023.1127320)
Supplement: Supplementary file 1 [file Table1.docx]

Supplementary table 1. Criteria for establishing the diagnosis of AMI in Kazakhstan

| **№** | **Description** |
| --- | --- |
| 1 | identification of an increase and / or a regular decrease in cardio specific enzymes (preferably troponin) in combination with at least one of the following criteria: |
| 2 | symptoms of myocardial ischemia; |
| 3 | diagnostically significant ST-segment elevation or newly registered blockade of the left bundle branch block; |
| 4 | pathological Q wave on the ECG; |
| 5 | the appearance of a non-viable myocardium or the identification of zones of hypo-/akinesis; |
| 6 | detection of intracoronary thrombosis during angiographic or post-mortem examination. |
| 7 | Cardiac death with symptoms suggestive of myocardial ischemia and prior ECG changes interpreted as ischemic, first reported LBBB, which occurred before the result of the analysis for myocardial necrosis markers, or occurred before the expected increase in their concentration in the blood. |
| 8 | PCI - associated MI is established on the basis of an increase in the concentration of troponin (> 5 × 99 percentile of ULN) in individuals with initially normal values (≤ 99 percentile of ULN), or an increase of 20% or more in cases where its level is initially elevated. Additionally, attention is paid to clinical signs of myocardial ischemia, new ischemic changes on the ECG, complications of coronary angiography, and detection of non-viable myocardium or atypical movement of the myocardial walls during echocardiography. |
| 9 | Stent thrombosis in myocardial infarction, detected during coronary angiography or pathoanatomical examination, in conditions of myocardial ischemia and an increase and / or subsequent regular dynamics of the level of cardio specific enzymes near the 99th percentile of ULN. |
| 10 | CABG-associated MI is defined as elevated troponin levels (>5 x 99 percentile ULN) in individuals with baseline normal values (≤99 percentile ULN). Additional criteria are: the appearance of an abnormal Q wave on the ECG, previously unrecorded LBBB, angiographically confirmed occlusion of the bypass or native coronary artery, and the detection of non-viable myocardium or atypical movement of the myocardial walls according to echocardiography. |

Supplementary table 2. Kazakhstan population by gender and age groups in 2021.

| **Age groups** | **Female** | **Male** | **Total** |
| --- | --- | --- | --- |
| 0-4 | 950 906 | 1 012 002 | 1 962 908 |
| 5-9 | 885 072 | 936 274 | 1 821 346 |
| 10-14 | 712 365 | 753 213 | 1 465 578 |
| 15-19 | 553 838 | 580 061 | 1 133 899 |
| 20-24 | 598 721 | 622 337 | 1 221 058 |
| 25-29 | 769 250 | 768 741 | 1 537 991 |
| 30-34 | 794 922 | 775 041 | 1 569 963 |
| 35-39 | 656 021 | 638 200 | 1 294 221 |
| 40-44 | 600 432 | 563 949 | 1 164 381 |
| 45-49 | 564 071 | 515 780 | 1 079 851 |
| 50-54 | 532 405 | 470 589 | 1 002 994 |
| 55-59 | 544 538 | 455 448 | 999 986 |
| 60-64 | 434 915 | 328 905 | 763 820 |
| 65-69 | 336 887 | 221 184 | 558 071 |
| 70-74 | 191 317 | 112 335 | 303 652 |
| 75-79 | 170 832 | 82 267 | 253 099 |
| 80-84 | 125 145 | 53 890 | 179 035 |
| 85-89 | 42 607 | 15 539 | 58 146 |
| 90-94 | 15 402 | 5 497 | 20 899 |
| 95-99 | 2 234 | 1 394 | 3 628 |
| **Total** | **9 481 880** | **8 912 646** | **18 394 526** |
